# Data on hepatitis B, C signs, and HIV-aids among middle-aged and older IDUs in Southwest Iran

**DOI:** 10.1016/j.dib.2018.02.046

**Published:** 2018-02-20

**Authors:** Leila Ghahremani, Eghbal Zarei, Hosein Moltafet, Abdolrahim Asadollahi

**Affiliations:** a Dept. of Health Education & Promotion, Faculty of Health, Shiraz University of Medical Sciences, Shiraz, Iran; b Dept. of Family Psychology, School of Humanity, University of Hormozgan, Bandar-Abbas, Iran; c Dept. of Sociology, School of Social Sciences, Shahid Chamran University of Ahvaz, Ahwaz, Iran; d Dept. of Aging Health, Faculty of Health, ShirazUniversity of Medical Sciences, Shiraz, Iran

## Abstract

Presented data was gathered from a local rehabilitation & caring center in Ahwaz city, southwest Iran end-2017. Data was classified in the six sections upon two predictors i.e. years of imprisonments and Injecting Drug Users (*IDUs*) to predict three blood infectious diseases i.e. HIV-Aids, Hepatitis B Virus (HBV), & Hepatitis C Virus (HCV). Each section jointly includes the following: tables of raw data, input data concerning false & true positive fractions (18 tables), scatter graph concerning false positive fractions (FPF) & true positive fractions (TPF) (6 graphs), and six ROC curves. The full access to data set is available at Mendeley data repository via doi:10.17632/6s9yzm7hr7.1.

**Specifications table**

**Table:** 

Subject area	*Health Sciences, Aging, Social Pathology, Prison Studies*
More specific subject area	*Hepatitis and HIV in Middle & Older Adults*
Type of data	*Table, text file, graph*
How data was acquired	*Field survey, Field investigation with residents, Review of reports*
Data format	*Raw & filtered*
Experimental factors	*Nil.*
Experimental features	*Based on patients' view & laboratory reports*
Data source location	*Ahwaz, Iran: 31*°*21*′*47.7"N & 48*°*44*′*05.6*″*E*
Data accessibility	*Brief data is presented with this article and the full access to data set is available at: Asadollahi, Abdolrahim (2018), “Hepatitis B, C Signs and HIV-Aids among Middle-Aged and Older IDUs in the Southwest Iran”, Mendeley Data, v1*http://dx.doi.org/10.17632/6s9yzm7hr7.1

**Value of the data**

• The data shows the health status of IDUs and prisoners in Ahwaz city, Iran.
• Data show the actual effectiveness and outcomes of imprisonments and IDU records in the health prisoners.
• The data helps to know the preferences of the HIV-Aids patients on the urban & prison settlements.
• The data helps to know the preferences of the HBV and HCV patients on the urban & prison settlements.
• Also, the dataset can help health care providers and policymakers in preventing and controlling diseases associated with blood infections in particular social group i.e. prisoners, refugees, gangs, vandals, addicts, & IDUs.

## 1 Data

Presented data classified in six sections upon two predictors i.e. years of imprisonments and IDU to predict three blood infectious diseases i.e. HIV-Aids, HBV, & HCV. Each section jointly includes the following: tables of raw data, input data concerning false & true positive fractions (18 tables), scatter graph concerning FPF & TPF (6 graphs), and six ROC curves using web-based calculator for ROC curves at www.jrocfit.org [1].

## 2 Experimental design, materials and methods

The cross-sectional method was done at the end-2017 in Ahwaz metropolitan, southwest Iran. About 133 injected drug abusers aged 40 to 71 years old (Mean age=48.21±10.4) were chosen from some addiction treatment centers. The data was collected from the setting in demographic and behavioral characteristics. Also, the serum samples were screened for those diseases. Their blood serum was evaluated for anti-hepatitis C, HIV-Aids, and antigen levels of hepatitis B by ELISA method. All positive cases from the ELISA analysis of HIV-Aids and HCV by immunoblot method were also evaluated (WB, Gene Labs Diagnostics, Ltd). The full data set is available online at Asadollahi, 2018 [2].

## 3 HBV & predictive variable years of IDU

**Fig. 1:**
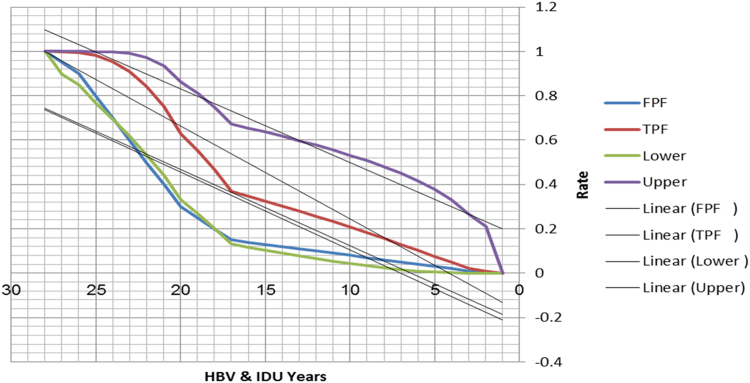
**Scatter Graph Concerning False & True Positive Fractions in Years of IDU and HBV**. Total Cases: 133, Positive Cases: 8, Negative Cases: 125, FPF: False Positive Fraction, TPF: True Positive Fraction, Fitted ROC Area: 0.731.

**Fig. 2:**
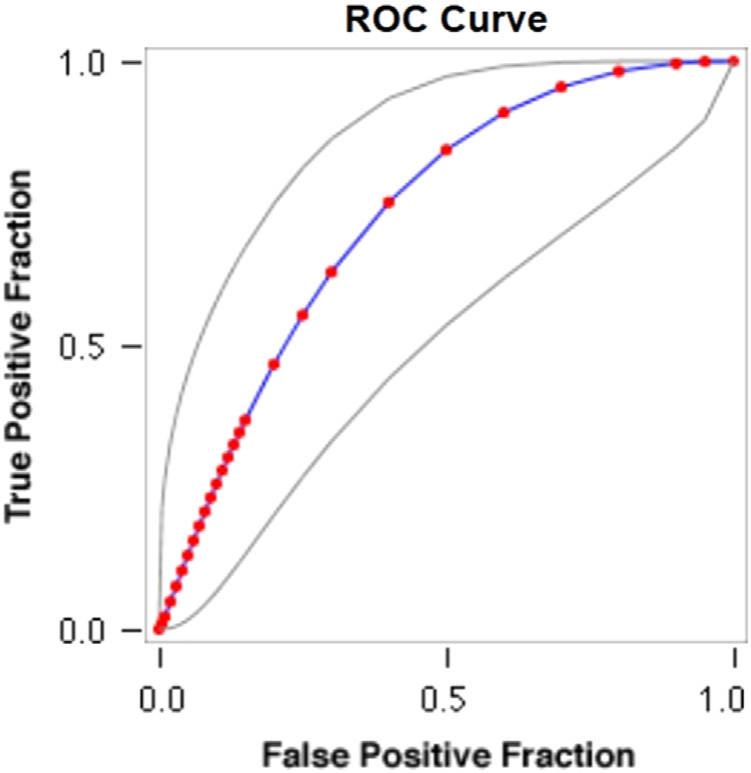
**ROC Curve Concerning FPF & TPF in Years of IDU and HBV**. Red symbols and Blue line: Fitted ROC curve, Gray lines: 95% confidence interval of the fitted ROC curve, Black symbols±Green line: Points making up the empirical ROC curve. Area Under Roc Curve: Area under fitted curve (Az)=**0.7309**, Estimated std. error=0.0793, Trapezoidal (Wilcoxon) area=0.7260, Estimated std. error=0.1043.

See Table 1, Table 2, Table 3 and Fig. 1, Fig. 2 here.

**Table 1:** Input data concerning years of IDU from the 125 actually negative HBV cases.

*Scores from the 125 actually negative cases:*				
8	6	6	18	18
17	18	15	21	21
22	19	20	20	8
18	17	13	17	19
18	19	17	1	22
18	19	18	5	6
17	0	12	0	0
8	3	14	0	10
0	0	0	7	0
0	0	0	0	0
0	16	17	0	0
0	13	10	10	16
7	15	17	13	0
19	17	12	0	17
0	16	16	0	11
0	16	23	15	18
20	10	9	8	17
10	7	9	20	18
17	10	8	13	7
16	17	19	7	18
18	9	22	9	17
18	23	17	14	21
20	20	21	19	16
15	16	9	19	17
17	21	3	21	3

*Scores from the 8 actually positive cases:* 8 17 17 22 16 21 20 20.

Larger values of the test result represent stronger evidence that the case is actually positive (e.g., that the patient is actually abnormal).

Number of actually negative cases=125.

Number of actually positive cases=8.

**Table 2:** Input data concerning false & true positive fractions in HBV & years of IDU.

**FPF**	**TPF**	**Lower**	**Upper**
0	0	0	0
0.005	0.0097	0.0001	0.2116
0.01	0.022	0.0003	0.2636
0.02	0.0485	0.0021	0.3301
0.03	0.0757	0.0053	0.3778
0.04	0.1028	0.0102	0.4166
0.05	0.1296	0.0166	0.4512
0.06	0.156	0.0243	0.4798
0.07	0.1818	0.0333	0.5069
0.08	0.2071	0.0434	0.5319
0.09	0.2318	0.0543	0.5553
0.10	0.2559	0.0659	0.5773
0.11	0.2795	0.0782	0.5981
0.12	0.3024	0.0913	0.6179
0.13	0.3248	0.1041	0.6368
0.14	0.3467	0.1176	0.6548
0.15	0.3679	0.1313	0.6722
0.22	0.4665	0.2006	0.749
0.25	0.553	0.2679	0.8121
0.31	0.6286	0.3306	0.8632
0.42	0.7519	0.4412	0.9343
0.50	0.8435	0.5351	0.9732
0.61	0.9095	0.6177	0.9912
0.74	0.9544	0.694	0.998
0.83	0.9822	0.7684	0.9997
0.91	0.9962	0.8481	1
0.95	0.9992	0.8965	1
1	1	1	1

**Table 3:** Estimated binormal Roc curve, with lower & upper bounds of the asymmetric 95% confidence interval for true-positive fraction at a variety of false-positive fractions.

**FPF**	**TPF**	**95% Conf. interval**
0.005	0.0097	(0.0001, 0.2116)
0.010	0.0220	(0.0003, 0.2636)
0.020	0.0485	(0.0020, 0.3301)
0.030	0.0757	(0.0053, 0.3778)
0.040	0.1028	(0.0102, 0.4166)
0.050	0.1296	(0.0166, 0.4500)
0.060	0.1560	(0.0243, 0.4798)
0.070	0.1818	(0.0333, 0.5069)
0.080	0.2071	(0.0434, 0.5319)
0.090	0.2318	(0.0543, 0.5553)
0.100	0.2559	(0.0659, 0.5773)
0.110	0.2795	(0.0782, 0.5981)
0.120	0.3024	(0.0910, 0.6179)
0.130	0.3248	(0.1041, 0.6368)
0.140	0.3467	(0.1176, 0.6548)
0.150	0.3679	(0.1313, 0.6722)
0.200	0.4665	(0.2006, 0.7490)
0.250	0.5530	(0.2679, 0.8121)
0.300	0.6286	(0.3306, 0.8632)
0.400	0.7519	(0.4412, 0.9343)
0.500	0.8435	(0.5351, 0.9732)
0.600	0.9095	(0.6177, 0.9912)
0.700	0.9544	(0.6940, 0.9980)
0.800	0.9822	(0.7684, 0.9997)
0.900	0.9962	(0.8481, 1.0000)
0.950	0.9992	(0.8965, 1.0000)

## 4 HBV & predictive variable years of imprisonment

**Fig. 3:**
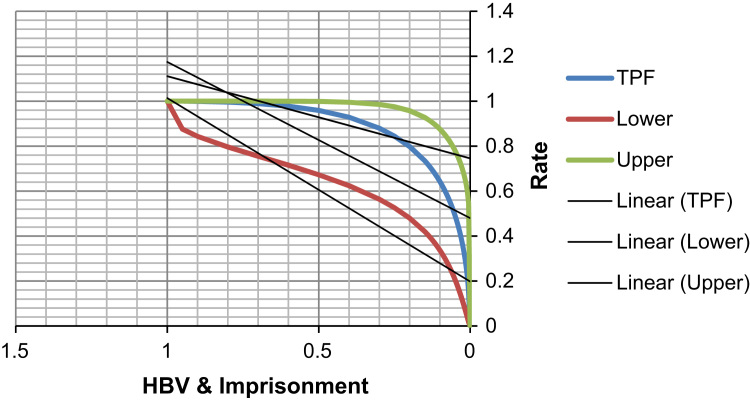
**Scatter Graph Concerning False & True Positive Fractions in Years of Imprisonment and HBV**. *Total Cases: 133, Positive Cases: 8, Negative Cases: 125, Fitted ROC Area: 0.882*.

**Fig. 4:**
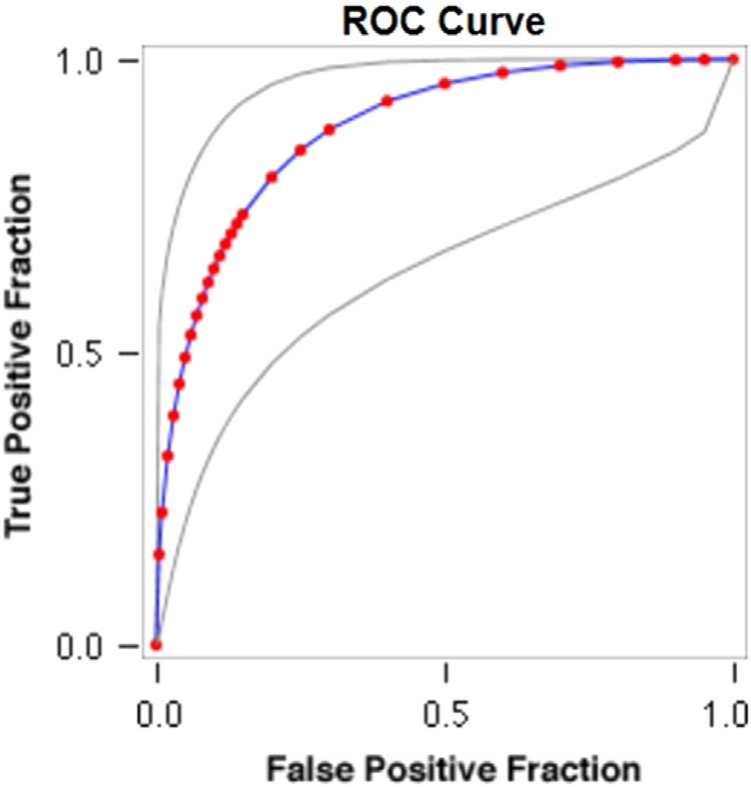
**ROC Curve Concerning FPF & TPF in Years of Imprisonment and HBV. Key for the ROC Plot**. Red symbols and Blue line: Fitted ROC curve, Gray lines: 95% confidence interval of the fitted ROC curve, Black symbols ± Green line: Points making up the empirical ROC curve. Area Under Roc Curve: Area under fitted curve (Az)=**0.8821**, Estimated std. error=0.0605, Trapezoidal (Wilcoxon) area=0.8760, Estimated std. error=0.0805

See Table 4, Table 5, Table 6 and Fig. 3, Fig. 4 here.

**Table 4:** Input data concerning years of imprisonment from the 125 actually negative HBV cases.

*Scores from the 125 actually negative cases:*				
0	0	7	0	0
9	7	7	0	0
0	6	5	7	0
5	0	0	0	0
0	5	2	2	7
2	7	5	2	5
6	5	7	1	4
5	5	7	2	3
4	4	2	6	7
2	6	8	4	2
4	5	4	6	4
7	2	6	2	4
6	4	6	5	5
4	4	4	4	5
4	11	6	5	7
6	6	2	4	6
6	1	6	5	5
7	7	4	3	7
5	4	3	11	3
8	7	5	8	8
4	7	7	7	3
5	8	8	15	3
8	4	5	7	5
4	7	4	11	4
8	11	8	3	3

*Scores from the 8 actually positive cases:* 7 7 10 6 12 7 13 21.

Larger values of the test result represent stronger evidence that the case is actually positive (e.g., that the patient is actually abnormal).

Number of actually negative cases=125.

Number of actually positive cases=8.

**Table 5:** Input data concerning false & true positive fractions in years of imprisonment and HBV.

**FPF**	**TPF**	**Lower**	**Upper**
0	0	0	0
0.005	0.1543	0.0157	0.5465
0.01	0.2262	0.0389	0.6029
0.02	0.3227	0.0869	0.6701
0.03	0.3916	0.1313	0.7156
0.04	0.4459	0.1711	0.751
0.05	0.4909	0.2066	0.7801
0.06	0.5293	0.2383	0.8047
0.07	0.5627	0.2667	0.826
0.08	0.5923	0.2923	0.8446
0.09	0.6188	0.3156	0.861
0.1	0.6427	0.3367	0.8754
0.11	0.6644	0.3562	0.8883
0.12	0.6842	0.374	0.8998
0.13	0.7025	0.3906	0.91
0.14	0.7193	0.4059	0.9192
0.15	0.7349	0.4203	0.9274
0.2	0.7985	0.48	0.9575
0.25	0.8449	0.5262	0.9752
0.3	0.8801	0.564	0.9857
0.4	0.9286	0.6242	0.9955
0.5	0.9587	0.6728	0.9988
0.6	0.9776	0.7155	0.9997
0.7	0.9892	0.7556	1
0.8	0.9958	0.7965	1
0.9	0.9991	0.8438	1
0.95	0.9998	0.8759	1
1	1	1	1

**Table 6:** Estimated binormal Roc curve, with lower & upper bounds of the asymmetric 95% confidence interval for true-positive fraction at a variety of false-positive fractions.

**FPF**	**TPF**	**95% Conf. interval**
0.005	0.1543	(0.0157, 0.5465)
0.010	0.2262	(0.0389, 0.6029)
0.020	0.3227	(0.0869, 0.6701)
0.030	0.3916	(0.1313, 0.7156)
0.040	0.4459	(0.1711, 0.7510)
0.050	0.4909	(0.2066, 0.7801)
0.060	0.5293	(0.2383, 0.8047)
0.070	0.5627	(0.2667, 0.8260)
0.080	0.5923	(0.2923, 0.8446)
0.090	0.6188	(0.3156, 0.8610)
0.100	0.6427	(0.3367, 0.8754)
0.110	0.6644	(0.3562, 0.8883)
0.120	0.6842	(0.3740, 0.8998)
0.130	0.7025	(0.3906, 0.9100)
0.140	0.7193	(0.4059, 0.9192)
0.150	0.7349	(0.4203, 0.9274)
0.200	0.7985	(0.4800, 0.9575)
0.250	0.8449	(0.5262, 0.9752)
0.300	0.8801	(0.5640, 0.9857)
0.400	0.9286	(0.6242, 0.9955)
0.500	0.9587	(0.6728, 0.9988)
0.600	0.9776	(0.7155, 0.9997)
0.700	0.9892	(0.7556, 1.0000)
0.800	0.9958	(0.7965, 1.0000)
0.900	0.9991	(0.8438, 1.0000)
0.950	0.9998	(0.8759, 1.0000)

## 5 HCV & predictive variable years of IDU

**Fig. 5:**
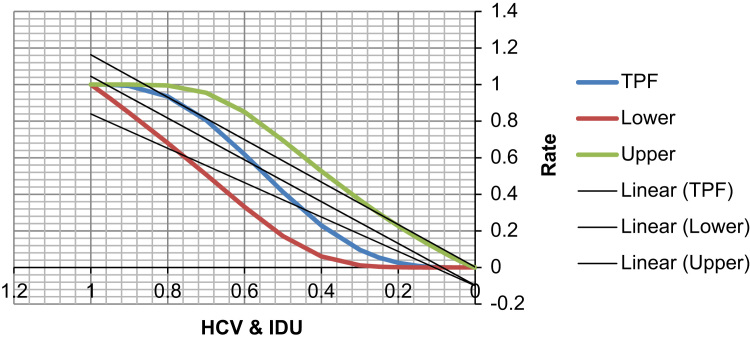
**Scatter Graph Concerning False & True Positive Fractions in HCV & Years of IDU**. *Total Cases: 133, Positive Cases: 11, Negative Cases: 122, Fitted ROC Area: 0.562.*

**Fig. 6:**
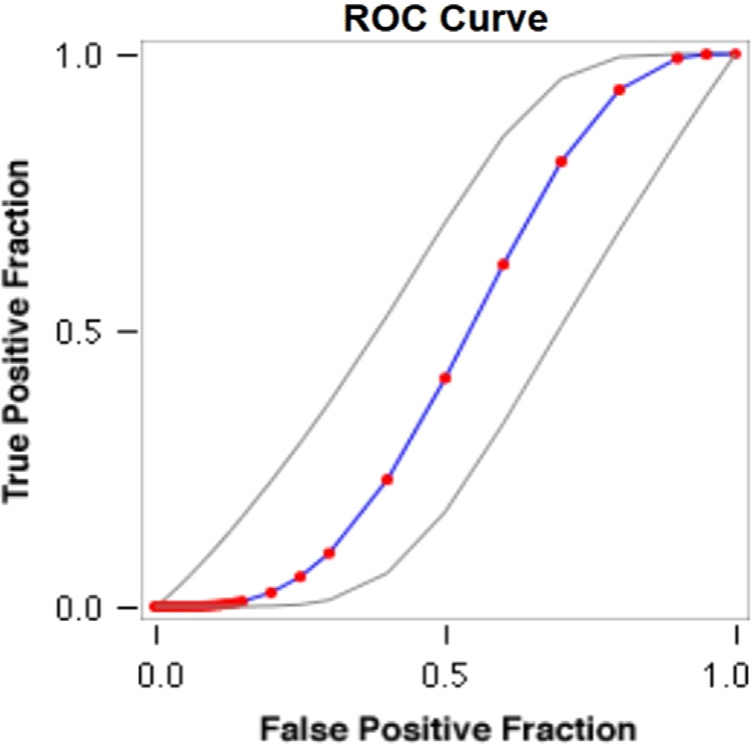
**ROC Curve Concerning FPF & TPF in HCV & Years of IDU**. Red symbols and Blue line: Fitted ROC curve, Gray lines: 95% confidence interval of the fitted ROC curve, Black symbols ± Green line: Points making up the empirical ROC curve. Area Under Roc Curve: Area under fitted curve (Az)=**0.4619**, Estimated std. error=0.0638, Trapezoidal (Wilcoxon) area=0.4694, Estimated std. error=0.0895.

See Table 7, Table 8, Table 9 and Fig. 5, Fig. 6 here.

**Table 7:** Input data concerning years of IDU from the 122 actually negative HCV cases.

*Scores from the 122 actually negative cases:*				
8	8	6	6	18
18	17	18	15	21
17	21	22	19	20
20	22	16	21	8
18	17	13	17	19
18	19	20	17	20
22	18	19	18	5
0	12	0	0	3
14	0	10	0	0
0	0	0	0	0
0	0	0	17	0
0	0	13	10	10
7	15	17	13	0
19	17	12	0	17
0	16	16	0	11
0	16	23	15	18
20	10	9	8	10
7	9	20	17	10
8	13	7	16	17
19	7	18	18	9
22	9	17	18	23
17	14	21	20	20
21	19	16	15	16
9	19	17	21	3
21	3			

*Scores from the 11 actually positive cases:* 7 16 16 17 18 17 1 6 17 8 17.

Larger values of the test result represent stronger evidence that the case is actually positive (e.g., that the patient is actually abnormal).

Number of actually negative cases=122.

Number of actually positive cases=11.

**Table 8:** Input data concerning false & true positive fractions in HCV & years of IDU.

**FPF**	**TPF**	**Lower**	**Upper**
0	0	0	0
0.005	0	0	0.0035
0.01	0	0	0.0076
0.02	0	0	0.0165
0.03	0	0	0.026
0.04	0.0001	0	0.0359
0.05	0.0002	0	0.0462
0.06	0.0003	0	0.0568
0.07	0.0006	0	0.0677
0.08	0.0009	0	0.0788
0.09	0.0014	0	0.0901
0.1	0.0021	0	0.1017
0.11	0.003	0	0.1134
0.12	0.0041	0	0.1253
0.13	0.0055	0	0.1374
0.14	0.0072	0.0001	0.1497
0.15	0.0092	0.0001	0.1622
0.2	0.0254	0.0008	0.2271
0.25	0.0538	0.0037	0.296
0.3	0.0969	0.0118	0.369
0.4	0.2295	0.0608	0.5269
0.5	0.4133	0.1715	0.6951
0.6	0.6189	0.3315	0.851
0.7	0.8055	0.5079	0.9557
0.8	0.9352	0.6806	0.9948
0.9	0.9923	0.8451	0.9999
0.95	0.9992	0.9244	1
1	1	1	1

**Table 9:** Estimated binormal Roc curve, with lower & upper bounds of the asymmetric 95% confidence interval for true-positive fraction at a variety of false-positive fractions.

**FPF**	**TPF**	**95% Conf. interval**
0.005	0.0000	(0.0000, 0.0035)
0.010	0.0000	(0.0000, 0.0076)
0.020	0.0000	(0.0000, 0.0165)
0.030	0.0000	(0.0000, 0.0260)
0.040	0.0001	(0.0000, 0.0359)
0.050	0.0002	(0.0000, 0.0462)
0.060	0.0003	(0.0000, 0.0568)
0.070	0.0006	(0.0000, 0.0677)
0.080	0.0009	(0.0000, 0.0788)
0.090	0.0014	(0.0000, 0.0901)
0.100	0.0021	(0.0000, 0.1017)
0.110	0.0030	(0.0000, 0.1134)
0.120	0.0041	(0.0000, 0.1253)
0.130	0.0055	(0.0000, 0.1374)
0.140	0.0072	(0.0001, 0.1497)
0.150	0.0092	(0.0001, 0.1622)
0.200	0.0254	(0.0008, 0.2271)
0.250	0.0538	(0.0037, 0.2960)
0.300	0.0969	(0.0118, 0.3690)
0.400	0.2295	(0.0608, 0.5269)
0.500	0.4133	(0.1715, 0.6951)
0.600	0.6189	(0.3315, 0.8510)
0.700	0.8055	(0.5079, 0.9557)
0.800	0.9352	(0.6806, 0.9948)
0.900	0.9923	(0.8451, 0.9999)
0.950	0.9992	(0.9244, 1.0000)

## 6 HCV & predictive variable years of imprisonment

**Fig. 7:**
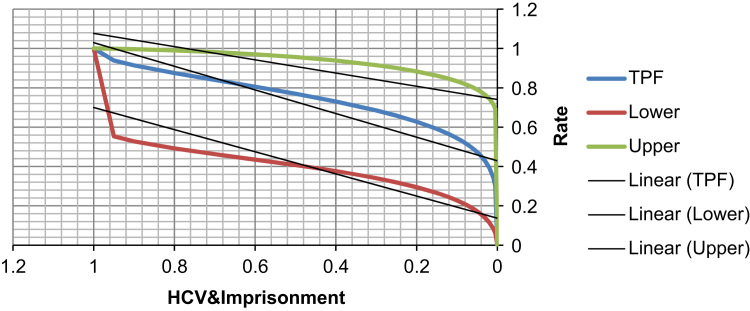
**Scatter Graph Concerning False & True Positive Fractions in HCV & Years of Imprisonment**. *Total Cases: 133, Positive Cases: 6, Negative Cases: 127, Fitted ROC Area: 0.746*.

**Fig. 8:**
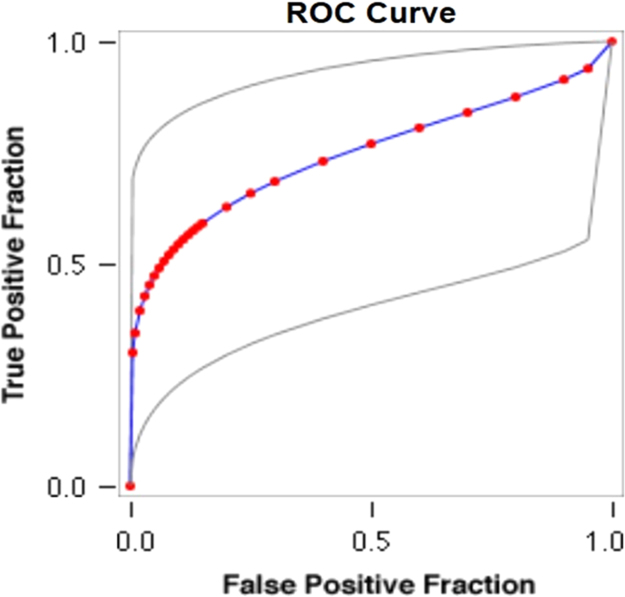
**ROC Curve Concerning FPF & TPF in HCV & Years of Imprisonment**. Red symbols and Blue line: Fitted ROC curve, Gray lines: 95% confidence interval of the fitted ROC curve, Black symbols ± Green line: Points making up the empirical ROC curve. Area Under Roc Curve: Area under fitted curve (Az)=**0.7463**, Estimated std. error=0.1355, Trapezoidal (Wilcoxon) area=0.7933, Estimated std. error=0.1113.

See Table 10, Table 11, Table 12 and Fig. 7, Fig. 8 here.

**Table 10:** Input data concerning years of imprisonment from the 127 actually negative HCV cases.

*Scores from the 127 actually negative cases*				
7	0	0	0	0
7	9	7	7	0
0	6	5	7	0
5	6	0	0	0
0	0	5	7	2
2	7	2	7	5
2	5	6	5	7
1	4	5	5	7
2	13	3	4	4
2	6	7	2	6
8	4	2	4	5
4	6	4	7	2
6	2	4	6	4
6	5	5	4	4
4	4	5	4	6
5	7	6	6	2
4	6	6	1	6
5	5	7	7	4
3	7	5	4	3
11	3	8	7	5
8	8	4	7	7
7	3	5	8	8
15	3	8	4	5
7	5	4	7	4
11	4	8	11	8
7	0	0	0	0

Scores from the 6 actually positive cases.

7 0 10 12 11 21.

Larger values of the test result represent stronger evidence that the case is actually positive (e.g., that the patient is actually abnormal).

Number of actually negative cases=127.

Number of actually positive cases=6.

**Table 11:** Input data concerning false & true positive fractions in HCV & years of imprisonment.

**FPF**	**TPF**	**Lower**	**Upper**
0	0	0	0
0.005	0.3001	0.0605	0.6922
0.01	0.3438	0.0843	0.7167
0.02	0.3942	0.1158	0.7451
0.03	0.4271	0.1385	0.7641
0.04	0.4523	0.1567	0.7788
0.05	0.4729	0.1721	0.7909
0.06	0.4905	0.1856	0.8014
0.07	0.5059	0.1976	0.8106
0.08	0.5197	0.2084	0.8189
0.09	0.5323	0.2183	0.8264
0.1	0.5438	0.2275	0.8333
0.11	0.5545	0.236	0.8397
0.12	0.5645	0.244	0.8457
0.13	0.5738	0.2515	0.8513
0.14	0.5827	0.2586	0.8566
0.15	0.5911	0.2654	0.8616
0.2	0.6277	0.2948	0.8832
0.25	0.6583	0.3193	0.9006
0.3	0.6849	0.3405	0.9153
0.4	0.7304	0.3764	0.9386
0.5	0.7698	0.4072	0.9565
0.6	0.8057	0.4353	0.9704
0.7	0.8401	0.4628	0.9814
0.8	0.875	0.4919	0.9899
0.9	0.9141	0.5275	0.9961
0.95	0.9387	0.5535	0.9984
1	1	1	1

**Table 12:** Estimated binormal Roc curve, with lower & upper bounds of the asymmetric 95% confidence interval for true-positive fraction at a variety of false-positive fractions.

**FPF**	**TPF**	**95% Conf. interval**
0.005	0.3001	(0.0605, 0.6922)
0.010	0.3438	(0.0843, 0.7167)
0.020	0.3942	(0.1158, 0.7451)
0.030	0.4271	(0.1385, 0.7641)
0.040	0.4523	(0.1567, 0.7788)
0.050	0.4729	(0.1721, 0.7909)
0.060	0.4905	(0.1856, 0.8014)
0.070	0.5059	(0.1976, 0.8106)
0.080	0.5197	(0.2084, 0.8189)
0.090	0.5323	(0.2183, 0.8264)
0.100	0.5438	(0.2275, 0.8333)
0.110	0.5545	(0.2360, 0.8397)
0.120	0.5645	(0.2440, 0.8457)
0.130	0.5738	(0.2515, 0.8513)
0.140	0.5827	(0.2586, 0.8566)
0.150	0.5911	(0.2654, 0.8616)
0.200	0.6277	(0.2948, 0.8832)
0.250	0.6583	(0.3193, 0.9006)
0.300	0.6849	(0.3405, 0.9153)
0.400	0.7304	(0.3764, 0.9386)
0.500	0.7698	(0.4072, 0.9565)
0.600	0.8057	(0.4353, 0.9704)
0.700	0.8401	(0.4628, 0.9814)
0.800	0.8750	(0.4919, 0.9899)
0.900	0.9141	(0.5275, 0.9961)
0.950	0.9387	(0.5535, 0.9984)

## 7 HIV-Aids & predictive variable years of IDU

**Fig. 9:**
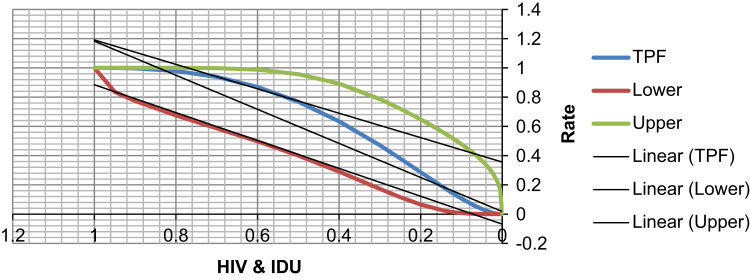
**Scatter Graph Concerning False & True Positive Fractions in HIV & Years of IDU**. *Total Cases: 133, Positive Cases: 6, Negative Cases: 127, Fitted ROC Area: 0.655*.

**Fig. 10:**
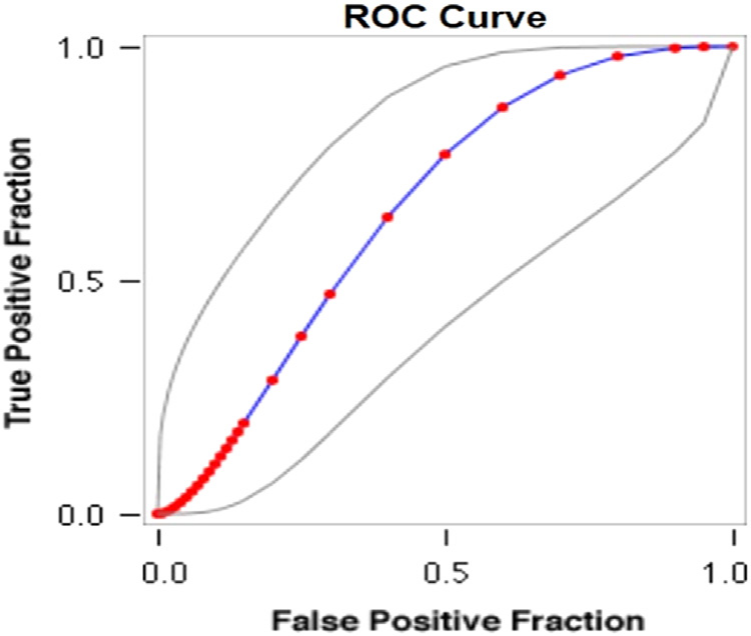
**ROC Curve Concerning FPF & TPF in HIV & Years of IDU**. Red symbols and Blue line: Fitted ROC curve, Gray lines: 95% confidence interval of the fitted ROC curve, Black symbols ± Green line: Points making up the empirical ROC curve. Area Under Roc Curve: Area under fitted curve (Az)=**0.6552**, Estimated std. error=0.0897, Trapezoidal (Wilcoxon) area=0.6483, Estimated std. error=0.1246.

See Table 13, Table 14, Table 15 and Fig. 9, Fig. 10 here.

**Table 13:** Input data concerning years of IDU from the 127 actually negative HIV cases.

*Scores from the 127 actually negative cases:*				
8	8	6	6	18
18	17	17	18	15
21	17	21	22	19
20	20	22	16	21
8	18	17	13	17
19	18	19	20	17
1	20	22	18	19
18	5	6	17	0
12	0	0	8	3
14	0	10	0	0
0	7	0	0	0
0	0	0	0	16
17	0	0	0	13
10	10	16	7	15
17	13	0	19	17
12	0	17	0	16
16	0	11	0	16
23	15	18	20	10
9	8	17	10	7
20	18	17	10	8
13	7	16	17	19
7	18	18	9	22
9	17	18	23	14
21	20	19	16	16
9	19	17	21	3
21	3			

Scores from the 6 actually positive cases: 9 17 20 21 15 17.

*Larger values of the test result represent stronger evidence that the case is actually positive (e.g., that the patient is actually abnormal).*

*Number of actually negative cases=127.*

*Number of actually positive cases=6.*

**Table 14:** Input data concerning false & true positive fractions in HIV & years of IDU.

**FPF**	**TPF**	**Lower**	**Upper**
0	0	0	0
0.005	0.0006	0	0.1691
0.01	0.0021	0	0.2112
0.02	0.0074	0	0.2658
0.03	0.015	0.0001	0.3054
0.04	0.0245	0.0002	0.338
0.05	0.0356	0.0005	0.3664
0.06	0.0479	0.0011	0.392
0.07	0.0613	0.002	0.4155
0.08	0.0757	0.0033	0.4376
0.09	0.0909	0.0051	0.4584
0.1	0.1068	0.0075	0.4783
0.11	0.1233	0.0104	0.4973
0.12	0.1403	0.014	0.5158
0.13	0.1577	0.0182	0.5336
0.14	0.1754	0.0231	0.551
0.15	0.1935	0.0286	0.568
0.2	0.2863	0.0658	0.6478
0.25	0.3798	0.1157	0.7206
0.3	0.4704	0.1729	0.7865
0.4	0.6346	0.2912	0.8922
0.5	0.7687	0.4002	0.9574
0.6	0.8697	0.4976	0.988
0.7	0.9386	0.5875	0.9979
0.8	0.979	0.6761	0.9998
0.9	0.9967	0.774	1
0.95	0.9995	0.8365	1
1	1	1	1

**Table 15:** Estimated binormal Roc curve, with lower & upper bounds of the asymmetric 95% confidence interval for true-positive fraction at a variety of false-positive fractions.

**FPF**	**TPF**	**95% Conf. interval**
0.005	0.0006	(0.0000, 0.1691)
0.010	0.0021	(0.0000, 0.2112)
0.020	0.0074	(0.0000, 0.2658)
0.030	0.0150	(0.0001, 0.3054)
0.040	0.0245	(0.0002, 0.3380)
0.050	0.0356	(0.0005, 0.3664)
0.060	0.0479	(0.0011, 0.3920)
0.070	0.0613	(0.0020, 0.4155)
0.080	0.0757	(0.0033, 0.4376)
0.090	0.0909	(0.0051, 0.4584)
0.100	0.1068	(0.0075, 0.4783)
0.110	0.1233	(0.0104, 0.4973)
0.120	0.1403	(0.0140, 0.5158)
0.130	0.1577	(0.0182, 0.5336)
0.140	0.1754	(0.0231, 0.5510)
0.150	0.1935	(0.0286, 0.5680)
0.200	0.2863	(0.0658, 0.6478)
0.250	0.3798	(0.1157, 0.7206)
0.300	0.4704	(0.1729, 0.7865)
0.400	0.6346	(0.2912, 0.8922)
0.500	0.7687	(0.4002, 0.9574)
0.600	0.8697	(0.4976, 0.9880)
0.700	0.9386	(0.5875, 0.9979)
0.800	0.9790	(0.6761, 0.9998)
0.900	0.9967	(0.7740, 1.0000)
0.950	0.9995	(0.8365, 1.0000)

## 8 HIV-Aids & predictive variable years of imprisonment

**Fig. 11:**
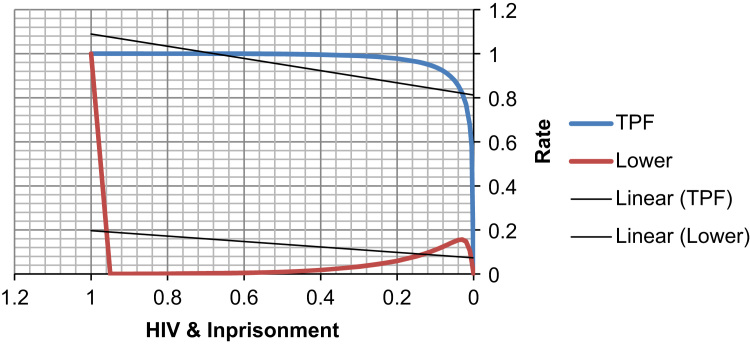
**Scatter Graph Concerning False & True Positive Fractions in HIV & Years of Imprisonment**. *Total Cases: 133, Positive Cases: 2, Negative Cases: 131, Fitted ROC Area: 0.977*.

**Fig. 12:**
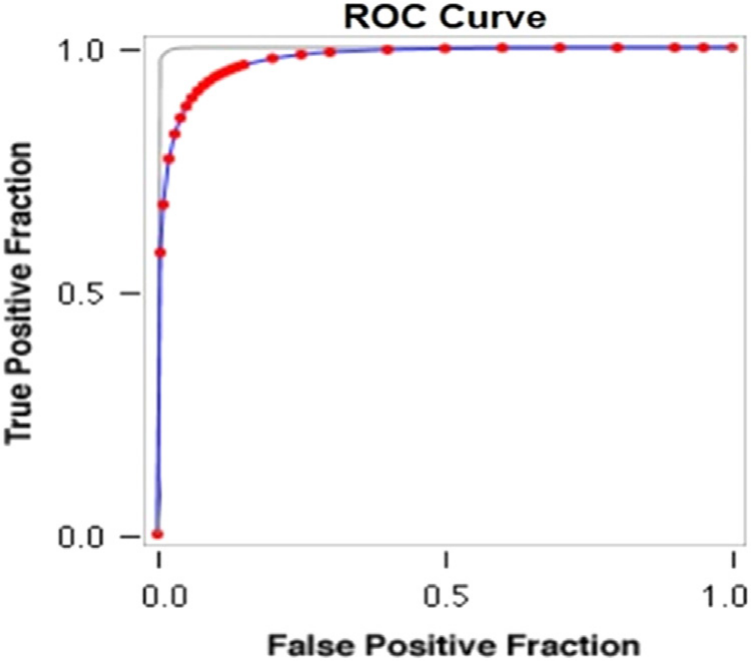
**ROC Curve Concerning FPF & TPF in HIV & Years of Imprisonment**. Red symbols and Blue line: Fitted ROC curve, Gray lines: 95% confidence interval of the fitted ROC curve, Black symbols ± Green line: Points making up the empirical ROC curve. Area Under Roc Curve: Area under fitted curve (Az)=**0.9771**, Estimated std. error=0.0483, Trapezoidal (Wilcoxon) area=0.9828, Estimated std. error=0.0649.

See Table 16, Table 17, Table 18 and Fig. 11, Fig. 12 here.

**Table 16:** Input data concerning years of imprisonment from the 131 actually negative HIV cases.

*Scores from the 131 actually negative cases:*				
7	0	0	7	0
0	7	9	7	7
0	0	0	6	5
7	0	10	5	6
0	0	0	0	0
12	5	7	2	2
7	2	7	5	2
5	6	5	7	1
4	5	5	7	2
13	3	4	4	2
6	7	2	6	8
4	2	4	5	4
6	4	7	2	6
2	4	6	4	6
5	5	4	4	4
4	5	4	11	6
5	7	6	6	2
4	6	6	1	6
5	5	7	7	4
3	7	5	4	3
11	3	8	7	5
8	8	4	7	7
7	3	5	8	8
15	3	8	4	5
7	5	4	7	4
4	8	11	8	3

Scores from the 2 actually positive cases: 11 21.

Larger values of the test result represent stronger evidence that the case is actually positive (e.g., that the patient is actually abnormal).

Number of actually negative cases=131.

Number of actually positive cases=2.

**Table 17:** Input data concerning false & true positive fractions in HIV & years of imprisonment.

**FPF**	**TPF**	**Lower**
0	0	0
0.005	0.5788	0.0553
0.01	0.6768	0.1068
0.02	0.7713	0.1481
0.03	0.8221	0.1565
0.04	0.8553	0.154
0.05	0.8789	0.1477
0.06	0.8968	0.14
0.07	0.9109	0.132
0.08	0.9222	0.1242
0.09	0.9315	0.1167
0.1	0.9393	0.1096
0.11	0.9459	0.1029
0.12	0.9515	0.0967
0.13	0.9564	0.0909
0.14	0.9607	0.0855
0.15	0.9644	0.0804
0.2	0.9777	0.0595
0.25	0.9855	0.0444
0.3	0.9903	0.0332
0.4	0.9956	0.0185
0.5	0.998	0.0099
0.6	0.9992	0.005
0.7	0.9997	0.0022
0.8	0.9999	0.0008
0.9	1	0.0001
0.95	1	0
1	1	1

**Table 18:** Estimated binormal Roc curve, with lower & upper bounds of the asymmetric 95% confidence interval for true-positive fraction at a variety of false-positive fractions.

**FPF**	**TPF**	**95% Conf. interval**
.005	0.5788	(0.0553, 0.9769)
.010	0.6768	(0.1068, 0.9847)
.020	0.7713	(0.1481, 0.9943)
.030	0.8221	(0.1565, 0.9979)
.040	0.8553	(0.1540, 0.9991)
.050	0.8789	(0.1477, 0.9996)
.060	0.8968	(0.1400, 0.9998)
.070	0.9109	(0.1320, 0.9999)
.080	0.9222	(0.1242, 1.0000)
.090	0.9315	(0.1167, 1.0000)
.100	0.9393	(0.1096, 1.0000)
.110	0.9459	(0.1029, 1.0000)
.120	0.9515	(0.0967, 1.0000)
.130	0.9564	(0.0909, 1.0000)
.140	0.9607	(0.0855, 1.0000)
.150	0.9644	(0.0804, 1.0000)
.200	0.9777	(0.0595, 1.0000)
.250	0.9855	(0.0444, 1.0000)
.300	0.9903	(0.0332, 1.0000)
.400	0.9956	(0.0185, 1.0000)
.500	0.9980	(0.0099, 1.0000)
.600	0.9992	(0.0050, 1.0000)
.700	0.9997	(0.0022, 1.0000)
.800	0.9999	(0.0008, 1.0000)
.900	1.0000	(0.0001, 1.0000)
.950	1.0000	(0.0000, 1.0000)
