# Supplementary material for: Data on hepatitis B, C signs, and HIV-aids among middle-aged and older IDUs in Southwest Iran
Source: Data Brief. 2018 Feb 20;17:1253–70. doi: 10.1016/j.dib.2018.02.046 (PMC5988449; doi:10.1016/j.dib.2018.02.046)
Supplement: Supplementary file 1 — Supplementary material [file mmc1.doc]

**COI Form**

**Authors' Contributions**

LGH contributed to the design, performed the interviews, and interpreted the data. HM has contributed to the design & interpretation. AA & EZ analyzed the data, wrote the draft and has revised the content, and scientific writing. All authors have approved the final manuscript.

**Source of Support:** Nil.

**Conflict of Interests:** The authors declare that they have no competing interests.
